# Insights for conservation from the Ecological Knowledge Games project

**DOI:** 10.1111/cobi.70308

**Published:** 2026-05-10

**Authors:** Yuan Pan, A. Bradley Duthie, Isabel Jones, Daksha Patel, Nils Bunnefeld

**Affiliations:** ^1^ Department of Digital Humanities King's College London London UK; ^2^ Biological and Environmental Sciences University of Stirling Stirling UK; ^3^ The Cultural Negotiation of Science (CNoS) Northumbria University Newcastle upon Tyne UK

**Keywords:** cultural engagement, digital conservation tools, digital games, participatory conservation, serious games, stakeholder engagement, video games, Compromiso cultural, conservación participativa, herramientas digitales de conservación, implicación de las partes interesadas, juegos con fines educativos, juegos digitales, videojuegos, 关键词: 数字游戏、电子游戏、严肃游戏、数字生态保护、文化参与、利益相关方参与、参与式保护

## Abstract

Environmental conservation research requires robust methods for collecting large‐scale behavioral data and engaging diverse stakeholders in decision‐making processes. We (Y.P., A.B.D., and N.B.) created EcoKnowGames (Ecological Knowledge Games), a transdisciplinary project that develops knowledge games for conservation science and data collection. We explored two existing knowledge games created by ecologists for the project: Power Up!, which collected over 57,000 player decisions on energy–biodiversity trade‐offs, and RESTORE, focused on ecosystem restoration scenarios. Building on these examples, EcoKnowGames integrates game development with ecological modeling to establish pathways from player decision‐making data to predictive socioecological models. We developed EcoKnowGames open‐source game builder tool in partnership with an established game studio, which allowed researchers to create custom knowledge games without extensive technical expertise. EcoKnowGames addresses key challenges in game‐based research, including privacy protection, participant consent, community engagement, and policy applications. This novel approach contributes to conservation research by providing empirically tested tools for integrating human behavior data into socioecological models and offers a complementary approach to traditional stakeholder engagement methods in decision‐making processes.

## Abstract (es)

Conclusiones para la conservación extraídas del proyecto «Ecological Knowledge Games»

**Resumen:**

La investigación en conservación del medio ambiente requiere métodos sólidos para recopilar datos de comportamiento a gran escala e involucrar a diversas partes interesadas en los procesos de toma de decisiones. Nosotros (Y.P., A.B.D. y N.B.) hemos creado EcoKnowGames (Juegos de Conocimiento Ecológico), un proyecto transdisciplinar que desarrolla juegos de conocimiento para la ciencia de la conservación y la recopilación de datos. Para el proyecto, analizamos dos juegos de conocimiento ya existentes creados por ecólogos: Power Up!, que recopiló más de 57 000 decisiones de los jugadores sobre las compensaciones entre energía y biodiversidad, y RESTORE, centrado en escenarios de restauración de ecosistemas. Partiendo de estos ejemplos, EcoKnowGames integra el desarrollo de juegos con la modelización ecológica para establecer vías que conecten los datos de las decisiones de los jugadores con modelos socioecológicos predictivos. Desarrollamos la herramienta de creación de juegos de código abierto EcoKnowGames en colaboración con un estudio de videojuegos consolidado, lo que permitió a los investigadores crear juegos de conocimiento personalizados sin necesidad de amplios conocimientos técnicos. EcoKnowGames aborda retos clave en la investigación basada en juegos, como la protección de la privacidad, el consentimiento de los participantes, la participación de la comunidad y las aplicaciones políticas. Este enfoque novedoso contribuye a la investigación sobre conservación al proporcionar herramientas, probadas empíricamente, para integrar datos de comportamiento humano en modelos socioecológicos y ofrece un enfoque complementario a los métodos tradicionales de participación de las partes interesadas en los procesos de toma de decisiones.

## 摘要

生态知识游戏) 项目对保护研究的启示

环境保护研究需要稳健的方法来收集大规模行为数据, 并促进多样化利益相关者参与决策过程。我们(Y.P, A.B.D., and N.B)创建了 EcoKnowGames(生态知识游戏)项目。这是一个跨学科项目, 致力于开发“知识游戏”, 以支持保护生物学中的社区科学(community science)实践与行为数据收集。我们分析了两款由生态学家开发的现有知识游戏:Power Up! (收集了超过 57,000 条玩家在能源与生物多样性权衡方面的决策数据)以及 RESTORE(聚焦生态系统恢复情景)。以此为基础, EcoKnowGames 将游戏开发与生态建模相结合, 建立了从玩家决策数据到预测性社会‐生态模型 的转化路径。通过与成熟的游戏工作室合作, 该项目开发了一个开源游戏构建工具, 使研究人员无需深厚的技术背景即可创建定制化的知识游戏。此外, EcoKnowGames 还解决了游戏化研究中的关键挑战, 包括隐私保护、知情同意、社区参与及政策应用。这一创新方法为保护研究提供了经实证检验的工具。通过知识游戏, 研究者可将人类行为数据纳入社会‐生态模型, 也为传统的利益相关者参与决策方式开辟了新的路径。

## INTRODUCTION

The unprecedented scale of global conservation challenges demands innovative approaches to engage diverse audiences and collect behavioral data at scales necessary to inform policy decisions (Veríssimo et al., 2024). Conservation research faces considerable obstacles that traditional methods struggle to address effectively.

Traditional community and citizen science approaches, although valuable, face significant limitations. For instance, most projects rely on episodic volunteer participation that produces fragmented datasets that do not capture decision‐making across diverse contexts well (Stosch et al., 2021). Digital games present opportunities to address these limitations through expanded participation mechanisms and automated data collection systems. With approximately 3.32 billion active gamers worldwide (PlayToday, 2023), conservation researchers could use digital games to reach wider demographics, including audiences disconnected from environmental discourse (Barab et al., 2010). Conservation and digital games are linked through initiatives like the UN Sustainable Development Goals Games Summit and the Playing for the Planet Alliance. Survey data from 380,000 digital gamers reveal that 81% want more environmental content in games and two thirds consider behavioral changes due to in‐game messaging (Playing4thePlanet, 2023).

We focused on digital knowledge games, defined by Schrier (2016) as “games that aim to produce new knowledge, solve real‐world problems, and/or generate new ideas and insights.” Schrier (2016) also contends that such “games use the power of crowdsourcing and collective intelligence, motivating many people to collaborate and solve complex problems through gameplay.”

Knowledge games draw on established theoretical frameworks from behavioral and human–computer interaction research. For example, construal level theory (Trope & Liberman, 2010) suggests that people struggle to connect with distant environmental problems. Games may reduce psychological distance through compressed timescales and direct interaction. These theoretical frameworks also highlight a critical design challenge: achieving ecological authenticity while maintaining cognitive accessibility. Design challenges might depend on environmental contexts, precluding universal solutions for balancing authenticity and accessibility.

Methodologically, knowledge games allow real‐time observation of decision‐making processes. This can reveal adaptive strategies through iterative ecosystem interactions while engaging larger participant pools and commonly excluded stakeholders like immigrants and youth (Publications Office of the European Union, 2025). Games can compress multidecadal ecological processes into observable time frames and create emotional connections through narratives (Ahn et al., 2016; Chang, 2017). However, the gap between game environments and real‐world complexity remains a challenge. Gaming environments cannot completely capture socioeconomic constraints, institutional barriers, and cultural factors influencing real‐world decisions.

Although previous studies have demonstrated digital games’ potential for environmental education and behavior change (Dunn et al., 2021; Fisher et al., 2021; Georgiou et al., 2023; Sandbrook et al., 2015), research into the wider capabilities of environmental knowledge games and frameworks for researcher–game developer collaboration remains limited. We explored outputs from the EcoKnowGames project, a transdisciplinary initiative developing knowledge games and exploring how they can advance conservation research. We considered practical way to translate research into effective game mechanics while addressing methodological challenges. This indicates that knowledge games can be complementary tools to traditional research approaches.

## PATHWAYS FROM KNOWLEDGE TO IMPACT

Conservation interventions typically fail not because of insufficient environmental data but because they rest on inaccurate assumptions about human behavior and stakeholder priorities (Veríssimo et al., 2024). The challenge is ensuring knowledge generation leads to tangible conservation outcomes. We examined how knowledge games contribute to conservation through three theoretically grounded pathways, each with clear mechanisms linking game‐generated data to conservation impact.

First, the behavioral pathway addresses the stated‐action gap that undermines conservation planning. Traditional questionnaire surveys frequently elicit socially desirable responses that may not reflect the constraints governing real decisions (Pan et al., 2020). Knowledge games act as virtual laboratories in that they use construal level theory principles (Trope & Liberman, 2010) to reduce psychological distance through concrete scenarios with immediate trade‐offs. Rather than asking abstract preference questions, games require players to allocate limited resources under realistic constraints, revealing actual decision thresholds. This generates revealed‐preference data (Costanza et al., 2014), which allow the design of behavior change interventions based on accurate stakeholder logic. By reducing psychological distance, games shift players from abstract deliberation to experiential learning.

Second, the scientific pathway improves predictive accuracy of socioecological models, which currently treat human behavior as static variables. We devised a parameterization loop in which game data inform empirical weights for social response functions in models. By exposing diverse demographics to systematically varied scenarios, researchers can identify social tipping points. When models accurately predict stakeholder responses to environmental changes or interventions, conservation researchers can optimize resource allocation and design more robust strategies.

Third, the policy pathway provides risk‐free scenario testing before costly implementation. Conservation policy failures are often irreversible. Once a protected area generates community conflict or if payment schemes rest on incorrect assumptions, conservation can be set back years. Drawing on participatory decision‐making principles (Reed, 2008), knowledge games democratize planning by allowing diverse stakeholders to engage with trade‐offs through interactive simulations. Games reveal unintended consequences, highlight resistance early, and build community buy‐in. This prevents failures from top‐down implementation.

These pathways operate synergistically: behavioral data parameterize scientific models; validated models allow more sophisticated policy testing; and policy experiments generate new behavioral data. This integration ensures knowledge generation serves conservation goals rather than distracting from them. Established knowledge games in diverse disciplines validate these mechanisms. FoldIt (a puzzle‐platformer game in which players manipulate 3D protein structures) solved intractable protein structures through human intuition (Cooper et al., 2010). Similarly, Quantum Moves demonstrated the potential for uncovering problem‐solving strategies that are not obvious through gameplay, challenging players to develop intuitive strategies for manipulating quantum states (Sørensen et al., 2016). Sea Hero Quest (a navigation game collecting player spatial navigation data) validates the feasibility of collecting large‐scale, cross‐cultural behavioral data while maintaining rigorous research standards and participant privacy. The data generated insights into establishing normative baselines for spatial memory (Coutrot et al., 2018).

These examples confirm that aligning game objectives with research goals and providing immediate feedback are essential design principles (Gee, 2003). This ensures that knowledge generation informs, rather than distracts from, real‐world decision‐making. The EcoKnowGames project uses these design principles to ensure that collected data and produced knowledge lead to more equitable and evidence‐based conservation outcomes.

## CASE STUDIES OF ENVIRONMENTAL KNOWLEDGE GAMES

The theoretical pathways discussed in the previous section are exemplified in two developed environmental knowledge games: Power Up! and RESTORE. These projects are more than just engagement or educational tools. They are virtual research environments that capture how people actually behave when they need to make difficult choices under specific constraints.

### Power Up!

**FIGURE 1:**
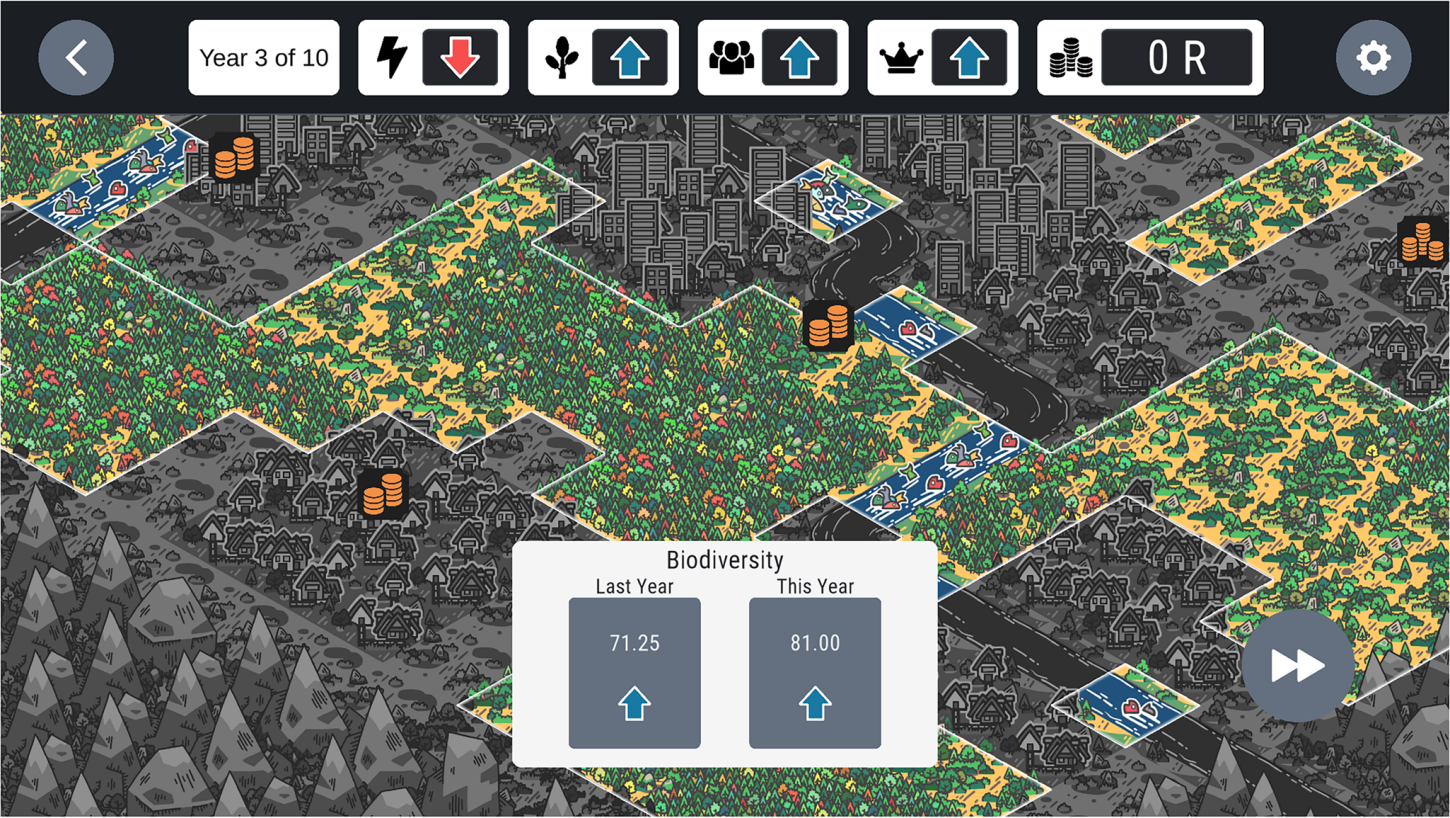
Screenshot from the environmental knowledge game Power Up! (I. Jones, personal communication). Players can see real‐time changes in the game landscape and in‐game resources based on resource allocation decisions and summaries (top bar) of the performance of the sustainable development goal metrics: renewable energy generation, biodiversity conservation, communities, and justice.

Power Up! (Figure 1) is a free mobile knowledge game developed by University of Stirling researchers and Hyper Luminal Games as part of the Beacon Project (Biodiversity, Energy Justice and Conflict), which models trade‐offs between UN Sustainable Development Goals (SDGs) in hydropower contexts. It provides a methodological lesson in the scalability of behavioral data collection. By recording over 57,000 player decisions regarding hydroelectric dam placement and player resource allocation to biodiversity conservation, communities, and energy generation (I. Jones, personal communication, 2026), the knowledge game has gathered a volume of data that would be impossible to achieve through traditional workshops or questionnaires. The value of these data is their ability to reveal how stakeholders balance competing SDG priorities (i.e., hydropower energy generation, biodiversity conservation, and community prosperity and justice) in real time. Conventional surveys often ask participants to rank these values in the abstract, but Power Up! has players face the immediate visual consequences of their choices. This provides researchers with a map of actual decision‐making logic, which can be used to create more accurate scientific models that predict how different groups might respond to SDG policies.

**FIGURE 2:**
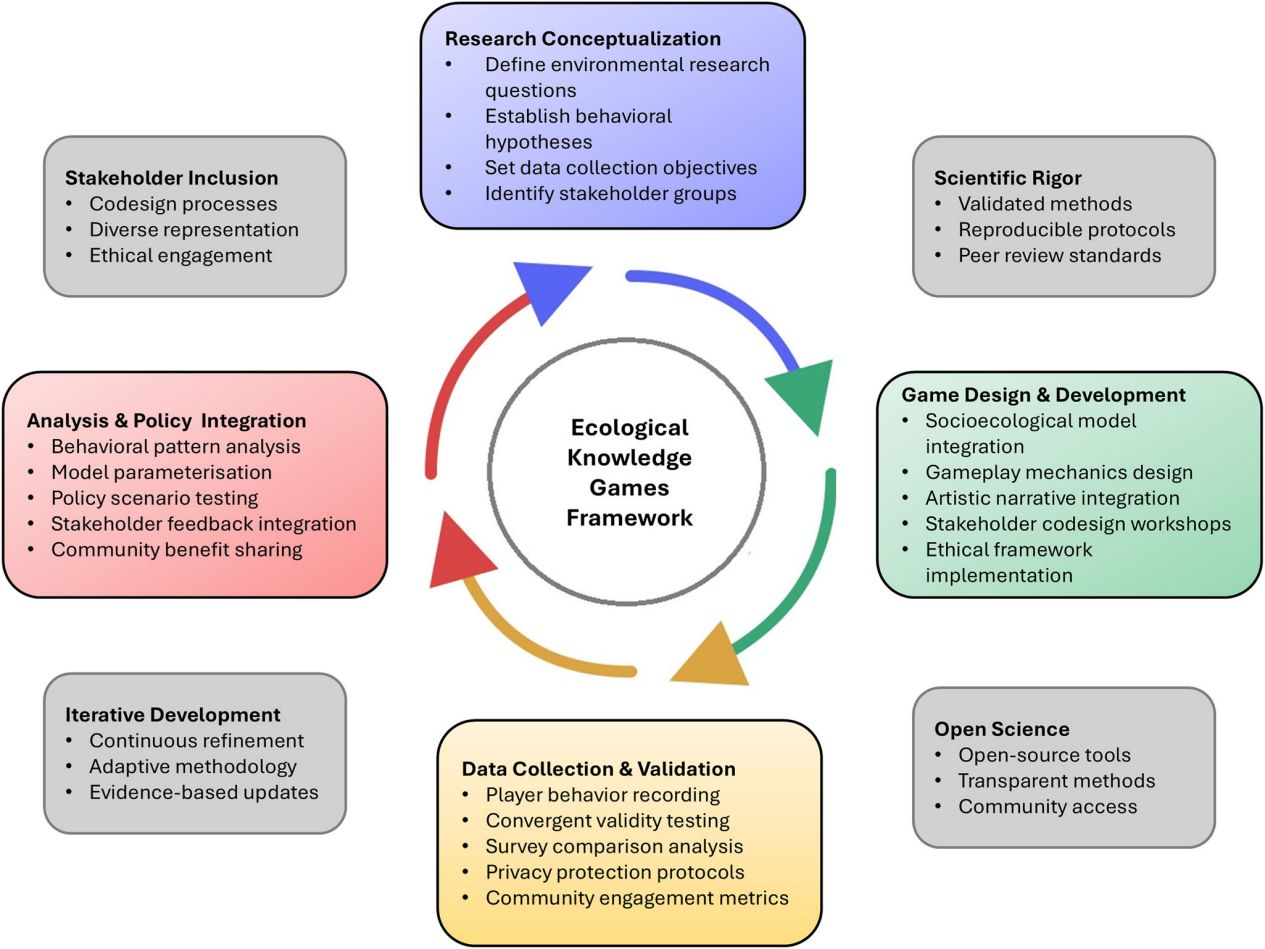
Iterative knowledge game development cycle. The central workflow (blue, green, yellow, and red) shows the operational phases of the EcoKnowGames framework, which are grounded in four foundational pillars (stakeholder inclusion, scientific rigor, iterative development, and open science).

### RESTORE

Although Power Up! demonstrates scale, RESTORE provides further insight into the complex and often surprising logic people use when managing landscapes. RESTORE is a free‐to‐play knowledge game developed by University of Stirling researchers with Glitchers (https://glitchers.itch.io/restore;). It was created as part of the RestoreID (Restoring Ecosystems to Stop the Threat of [Re‐] Emerging Infectious Diseases) project, a research program dedicated to exploring the links between nature restoration, biodiversity, and disease prevention. The player must balance competing land‐use demands while navigating the links between ecological restoration and disease spillover risk.

RESTORE highlights the point at which a player's behavior contradicts their stated values due to immediate pressure. For example, conventional conservation research might show that people generally support planting trees. However, RESTORE revealed the specific point at which a player might stop planting trees to avoid financial loss or the risk of disease. Some players chose to let their virtual populations go hungry because the game's penalties for a disease outbreak felt more threatening than the lack of food. Such knowledge is vital because it highlights tipping points where a conservation plan might fail in the real world due to human priorities. Furthermore, it demonstrates a critical methodological advantage. Knowledge games can reveal deep‐seated fears and economic trade‐offs that participants might be reluctant to admit in an interview. By using these data to improve scientific models, researchers can better predict how different groups will react to new environmental policies. These insights are transferable to other conservation contexts (e.g., managing forest fires or invasive species) in which human fear and economic survival often override long‐term environmental goals. Knowledge games turn stakeholder engagement into a more active process where community members help test and refine the strategies that will affect their own environments.

## METHODOLOGY OF EcoKnowGames

### Frameworks for game design and models underlying games

For the EcoKnowGames project, we used an integrated framework that links modeling, game development, and data collection. This framework addresses the technical challenges of creating knowledge games that maintain scientific rigor while providing engaging gameplay experiences (Figure 2).

General integration of scientific models and games requires a high level of modeling abstraction. If a modeling approach is too specialized to a particular system, this will limit the range of scientifically valid knowledge games that can be created within our framework. We therefore opted for a generalized Lotka–Volterra approach, which can accommodate a wide range of complex systems (May, 1972). In this approach, the change in density of any entity *i* can be affected by the density of any other entity *j* such that the per capita effect of *j* on *i* is *a_ij_*. Entities are traditionally interpreted as biological species but can be interpreted more broadly as any social or ecological variables in a complex system (e.g., Haldane & May, 2011). Interactions between *n* entities can be organized in an *n* × *n* square interaction matrix (**A**) in which off‐diagonal elements model interaction coefficients and diagonal elements model self‐regulation (e.g., intraspecific competition in an ecological community). Each entity has a growth rate **r** *_i_*, where **r** is a growth rate vector with *n* elements. At time *t*, the density of each entity is defined by the vector **N** *_t_*, and **N** *_t_*_ + 1_ is calculated as **N** *_t_*_ + 1_ = **N** *_t_* + **N** *_t_* (**r **+ **AN** *_t_*).

Our modeling framework includes a spatially explicit landscape organized into an arbitrary number and spatial pattern of discrete cells. Each cell has its own potentially unique density of entities, and within‐cell density change between time steps is defined by the above equation. Between time steps, entities may disperse among cells according to defined movement rules. For example, individual entities might disperse to a randomly selected adjacent cell with a species‐specific probability *d_i_*. This general modeling framework makes it possible to model a wide range of social–ecological systems with interacting components and potential spatial effects.

### EcoKnowGames tool

Accessible tools that effectively bridge the divide between complex environmental modeling and intuitive game design are lacking. We developed the EcoKnowGames tool to address this limitation and provide an integrated platform for designing and using knowledge games.

The tool's architecture is designed to accommodate a diverse user base through multiple accessibility levels. For nontechnical users, the primary interface allows for game creation without extensive programing expertise. This aspect makes the tool suitable in nontechnical settings, such as educational modules or capacity building courses for policy makers. Advanced users can modify the fundamental code through a GitHub repository (https://github.com/EcoKnowGames/creator). This repository allows collaborative development through discussion forums (https://github.com/orgs/EcoKnowGames/discussions), artwork contributions, and direct code modification.

A core component of the EcoKnowGames architecture is its robust, built‐in data collection capability. The system can record all participant decisions and log the value of every landscape entity throughout the simulation. These comprehensive data are saved in accessible formats optimized for subsequent statistical analysis. Although default logging is exhaustive, potentially creating large datasets, the architecture anticipates the need for data refinement. It is designed to support flexible filtering systems, allowing researchers to efficiently extract relevant data specific to their research questions and exclude irrelevant information.

### Integration between models and games

Integrating models with interactive game environments can contribute to multiple goals, for example, scientific modeling embedded in games, enhanced interaction with policy makers, and cocreation with stakeholders. Games may be less scientifically precise than pure models but offer greater control over player engagement and policy communication.

Game‐generated data can be integrated with models to improve the realism of environmental decision‐making simulations (Villamor et al., 2023). For example, the rich social–ecological environments found in many games are the foci of complex social–ecological models. These models have an ecological component (e.g., a resource that needs to be managed but might be affected by environmental variables and landscape‐level processes) and a social component (Schlüter et al., 2012). Hence, they need to simulate how decision makers and stakeholders will act in any given modeled situation. This might be something as simple as setting a harvest quota based on a current population density, but models targeting specific systems might also need to simulate decision‐making under conditions in which there are multiple competing management goals and uncertain sources of information. Knowledge games can become sustainable platforms for ongoing data collection that can inform environmental decision‐making (Smith et al., 2015; Valero et al., 2025). If in‐game conditions for player decision‐making align with the social component of social–ecological models, data collected from games could be applied to parameterizing complex models. The result could be a digital games‐based research program, in which stakeholders contribute to the parameterization of complex social–ecological models by playing complementary social–ecological games (Duthie et al., 2021).

By subjecting different groups of players to different in‐game decision‐making scenarios, researchers could test how players with different backgrounds and lived experiences differ in their decision‐making. In this way, knowledge games can act as platforms for participatory decision‐making (Costanza et al., 2014; Shakeri, 2016). This inclusive approach ensures that environmental strategies reflect the needs of communities.

### Ethical framework

EcoKnowGames addresses the unique challenge of collecting scientifically valid behavioral data through gaming platforms. Obtaining and maintaining informed consent in gaming environments presents distinct ethical considerations due to the continuous involvement of human participants. The interactivity of games can blur the boundaries between entertainment and data collection, potentially leading participants to forget they are contributing to research (Vayena et al., 2016; Wood et al., 2004). We followed clear ethical standards and processes for data collection that align with the Code of Practice for Research, the key principles of ethical research established in the Economic and Social Research Council Framework for Research Ethics, and the Association of Internet Researchers guidelines (Franzke et al., 2020). EcoKnowGames includes clear documentation with background, study aims, how the data will be used, the rights participants hold over their data, and a comprehensive privacy policy. Before gameplay starts, participants must provide recorded informed consent to participate. This information will be presented in accessible language to ensure participants fully understand their engagement as both players and research subjects.

A key component of our ethical design is the data‐sharing mechanism. Game data are not collected automatically or passively. Instead, data generated during gameplay are stored locally on the player's device. After completing the game, participants are actively asked to share their data for research. Data submission requires a specific and voluntary action from the player (clicking a “Save and Share” button), at which point the game data (in JavaScript object notation format) are transmitted. Players can choose not to take this action, in which case no data are collected by the research team. This process provides players with final control over their data after their game experience is complete. A participant can opt out at any point by closing the game or by declining to share the data at the end.

Once data are submitted, robust measures are applied to protect player confidentiality. To minimize the risk of reidentification, we focused on advanced anonymization techniques. We used multiple layers of data protection, such as the removal of all personally identifiable information and the aggregation of potentially sensitive variables. All collected data are securely managed according to the EU's General Data Protection Regulation (GDPR) requirements using encrypted, access‐controlled storage systems with regular backups. All code for extracting raw game data and curating them for analyses is version controlled and published as open‐source R packages that are publicly available through the Comprehensive R Archive Network (CRAN) and GitHub.

### Community engagement strategy

Our centering‐the‐margins approach prioritizes accessibility, equity, and inclusion throughout game development (Doucet, 2019; Hill Collins, 2002). This framework, rooted in feminist and social theory, centers the perspectives of communities historically excluded from environmental research and game development processes. By prioritizing marginalized voices, we aimed to create more equitable and representative research outcomes. This approach recognizes that those most affected by environmental decisions often have the least voice in research and policymaking (Publications Office of the European Union, 2025).

EcoKnowGames uses a multinetwork recruitment approach through partnerships with established institutions. We engaged conservation organizations and environmental policy networks via the Alternet–Eklipse network (an autonomous science–policy interface mechanism [https://eklipse.eu/]) and reached gaming communities through Discord (a free communication platform popular with gamers) servers and organizations, such as Women in Games and POC in Play (Reed, 2008; Shirk et al., 2012). We worked with local champions and stakeholder networks to navigate potential barriers to participation (Valero et al., 2025). This strategy engaged participants typically disconnected from environmental research and stakeholders directly affected by environmental decisions (Bonney et al., 2016). This approach allowed us to demonstrate concrete examples of our work while inviting early community feedback.

Stakeholder engagement occurred throughout development, testing, and refinement phases through workshops combining game‐based activities with traditional consultation methods (Cornwall & Jewkes, 1995; Pretty, 1995). These sessions included logistical support, such as venue provision, catering, and travel assistance, to reduce participation barriers. When the EcoKnowGames prototype is released, we will conduct stakeholder surveys comparing in‐game decision patterns with established environmental attitude measures (Schwartz, 2012; Stern et al., 1999; Valero et al., 2025). Community benefit sharing ensures participants receive research findings through summary reports and project website updates, addressing concerns about extractive research practices while building sustained engagement (Tuhiwai Smith, 2021).

### Artistic design principles

Environmental knowledge games require careful balancing of scientific rigor with engaging gameplay. Artistic elements, such as narrative structure, visual metaphors, and interactive storytelling, serve methodological functions. This can increase participant retention and create emotional investment that sustains long‐term engagement (Connolly et al., 2012).

Knowledge games represent a convergence of artistic expression and scientific inquiry, combining computing science, social science, psychology, and design principles. This creates opportunities for holistic approaches to understanding human–nature relationships. Environmental art theorists have long recognized art's capacity to reframe human–nature relationships (Brady & Prior, 2020). Knowledge games extend this capacity by combining artistic representation with scientific simulation, creating “playable models” of ecosystems (Sicart, 2017). This addresses a key challenge to link scientific understanding with emotional engagement (Carlson, 2000).

The artistic elements of digital games create distinctive pathways for environmental learning. Players become active participants, experiencing the consequences of their choices and creating “embodied environmental learning” (Ouariachi et al., 2017). Games transform abstract environmental data into experiential knowledge, which could improve player understanding of environmental processes (Ahn et al., 2016). Unlike traditional forms of passive environmental education, digital games actively engage players through creative storytelling and interactive experiences (Flanagan, 2013).

In our game development process, visual design directly supports research objectives. Artistic choices enhance players’ connection with ecosystems through visual metaphors representing ecological relationships or mapping techniques that reveal ecosystem changes (Fernández Galeote et al., 2023).

## CHALLENGES AND SOLUTIONS

### Game engine selection

A key challenge of game development is selecting an appropriate digital game engine (Table 1). Environmental researchers have options that require less technical expertise than building a game entirely from scratch or using other high‐end professional engines. The Godot Engine and Unity are suitable due to their ease of use, performance, and affordability. Godot Engine's open‐source nature, highly customizable architecture, and low system requirements make it a good platform for creating games that model environmental challenges. Unity's extensive community, vast asset store, and cross‐platform support make it a good choice for developing games that simulate complex environmental systems or promote sustainability awareness. We chose Unity as EcoKnowGames’ engine for the above reasons.

**TABLE 1:** A comparison of advantages, disadvantages, and pricing models of three major game development engines (Unity, Unreal Engine, and Godot) as of 2023.

Game engine	Advantages	Disadvantages	Pricing model
Unity	Cross‐platform support, large community and asset store, easy to learn	Performance issues with complex scenes, resource intensive	Free (small projects), subscription based (larger projects)
Unreal Engine	High‐performance capabilities, stunning visuals, large community, cross‐platform support	Steep learning curve, resource intensive	Royalty model (5% of gross revenue after $3000 per product per quarter)
Godot Engine	Open‐source and free, highly customizable and extensible architecture, small footprint, and low system requirements	Smaller community, less extensive resources	Free

These platforms integrate a suite of built‐in options for environmental knowledge game design, allowing the creation of interactive ecosystem features, such as dynamic landforms and vegetation. They also have robust data collection capabilities to systematically track and log player decisions and strategic choices. To convey the outcomes of these actions, the platforms use dynamic visualization tools that render environmental changes through compelling graphical feedback. In addition, these systems are often equipped for community engagement by incorporating multiplayer functionalities specifically designed to support participatory decision‐making processes.

To ensure long‐term impacts, ongoing updates need to reflect emerging environmental challenges, incorporate new data, and address player feedback. This requires a sustainable plan for digital game maintenance. Academic funding is often not long enough for ongoing game maintenance, and researchers might need to look further for other sources of funding, such as private funding or crowdsourcing funding.

### Validation against traditional methods

There are different validation methodologies for game‐generated data. The standard involves direct comparison between game outputs and established scientific methods. Environmental knowledge games face validation challenges because optimal environmental behaviors cannot be tested directly in controlled conditions. Therefore, this requires alternative validation strategies. Convergent validity involves demonstrating that game‐based measurements produce similar results to established survey methods when measuring the same environmental attitudes or behaviors. Predictive validity requires showing that models informed by game data can accurately forecast real‐world environmental outcomes. We plan to conduct pregame and in‐game surveys that allow validation against established environmental attitude scales (Dunlap et al., 2000; Rakotonarivo et al., 2021b; Stern et al., 1999). Survey data complement game workshop data to evaluate how values expressed by players in in‐game decisions reflect the values inferred from established survey methods (Schwartz, 2012). This approach builds on established practices in citizen science validation while addressing the challenges of game‐based research (Follett & Strezov, 2015; Kosmala et al., 2016).

### Limitations and appropriate applications

Knowledge games present some methodological constraints that restrict their application across conservation research. Hybrid methodologies can address some constraints, whereas others are inherent to game‐based research. First, knowledge games cannot address policy contexts that require extended stakeholder deliberation (Reed, 2008). The compressed time frames and predetermined choice set in game design eliminate the reflective deliberation needed for complex environmental governance.

In‐person gaming workshops partially overcome time compression issues by incorporating reflection periods. Workshop facilitators can pause gameplay for stakeholder discussion, and multisession workshops allow participants to reconsider decisions between sessions. These measures cannot fully replicate long‐term consultation. Knowledge games provide useful initial engagement, but complex policy contexts will always require traditional deliberative processes for final decision‐making (Reed, 2008)

Second, gaming populations skew toward younger, more educated, and technologically literate (Yee, 2006). This demographic bias is problematic for rural environmental conflicts because it risks excluding key stakeholders (e.g., elderly, low‐income, or Indigenous groups) who hold critical local knowledge but may lack digital experience or prefer different consultation protocols. These limitations may stem from recruitment strategy rather than the method itself. The ConFooBio project, for example, demonstrated that proactive recruitment could successfully engage rural, older, and low‐income stakeholders with minimal gaming experience, proving these biases can be overcome (Rakotonarivo et al., 2021a).

Community‐based gaming workshops can address technological barriers by providing facilitators and technical support in familiar environments (e.g., community centers or libraries). For example, taking tablets to locations where access to technology is limited or using board game versions of digital games eliminates technology requirements while maintaining core decision‐making elements. Furthermore, providing compensation and transportation removes participation barriers. However, people from cultures that view individual decision‐making as inappropriate may reject gaming approaches regardless of modifications.

Third, game environments necessarily reduce environmental complexity to tractable variable numbers. Real ecosystems involve thousands of interacting variables across temporal and spatial scales that exceed computational representation (Schlüter et al., 2012). Socioecological systems embed cultural values and power dynamics that are challenging to simulate. The constructed nature of games embeds developer assumptions that may contradict local ecological knowledge or community priorities.

Knowledge games excel at collecting standardized behavioral data across large and geographically dispersed populations. They allow rapid scenario testing that traditional methods cannot achieve at scale. However, ethnographic approaches and participatory research provide a deeper understanding of stakeholder motivations. Knowledge games cannot replace traditional methods, but they can make stakeholder engagement more interactive.

## POLICY APPLICATION

Conservation policy decisions often occur in complex socioecological contexts that have competing stakeholder interests, scientific uncertainty, and limited resources (Núñez‐Regueiro et al., 2020). Traditional approaches to informing these decisions can fail to capture the complexity of decision‐making or engage stakeholders effectively. Surveys and questionnaires typically rely on stated preferences, which often differ from actual behavior when people face real trade‐offs and constraints (Levitt & List, 2007). Expert elicitation, although valuable for technical knowledge, may not capture how nonexperts weigh different values and priorities (Krueger et al., 2012). Furthermore, traditional consultation processes have low participation rates, particularly among marginalized groups who may distrust formal institutional processes (Sarkki et al., 2024). Knowledge games could better inform conservation policy by collecting richer behavior data, testing policy scenarios, and building public support.

Policies fail when they make incorrect assumptions about human behavior (Sullivan‐Wiley et al., 2023). Knowledge games can provide data about how different stakeholders make decisions, helping policy makers design more effective interventions. Unlike surveys that ask what people would do hypothetically, games reveal what people do when facing realistic scenarios with trade‐offs, time pressures, and limited information. These are conditions that mirror real‐world decision‐making (Castilla‐Rho et al., 2017). By identifying behavioral patterns across different player demographics, knowledge games can help policy makers target interventions to specific groups.

Equally important to understanding behavior is improving stakeholder participation. Participatory processes are essential for effective policy, but achieving meaningful engagement remains challenging (Sterling et al., 2017). Knowledge games can transform stakeholder engagement by making complex environmental information accessible, allowing participants with varying levels of technical knowledge to engage with scientific concepts (Voinov et al., 2016). The structured format ensures all voices can be heard without being dominated by assertive participants. Multiplayer games create safe spaces to explore sensitive issues and provide opportunities to learn from each other's approaches, developing collective intelligence. This collaborative approach is valuable in contexts with historical conflicts, such as between researchers and Indigenous communities (Pan et al., 2025). Knowledge games can create neutral spaces for dialogue without power imbalances.

Moving from understanding to action, proposed conservation policies can be tested in knowledge games. Digital games compress timescales, allowing players to observe potential long‐term consequences of policy decisions. For example, a game simulating different protected area management strategies could demonstrate how different enforcement levels, community involvement approaches, and funding mechanisms might affect biodiversity over decades, which is all observable within hours of gameplay (Rakotonarivo et al., 2021a).

## CONCLUSION

The EcoKnowGames project expands the methodological toolkit available to conservation researchers through systematic development of knowledge games. The open‐source game builder tools (available at https://github.com/EcoKnowGames) create accessible pathways for researchers to adopt knowledge game approaches. This allows users to design and analyze games tailored to their specific conservation contexts.

Knowledge games are most effective when they generate complementary data at scales difficult to achieve through conventional approaches; however, they have limitations, such as validity constraints and context‐dependent effectiveness. Knowledge games cannot replace established participatory methods; rather, they enhance them through targeted community involvement.

Future advancement depends on the strategic integration of machine learning to identify complex behavioral patterns and refine predictive modeling for policy scenario outcomes. Sustained community engagement requires institutional partnerships with environmental organizations and policy bodies and exploration of alternative distribution models.

Success depends on continued collaboration among researchers, game developers, and affected communities. This must be coupled with clear awareness of appropriate application contexts and limitations. We invite researchers and practitioners to explore the toolkit and contribute to its evolution and to help determine whether knowledge games are a suitable conservation research method.

## AUTHOR CONTRIBUTIONS

**Yuan Pan**: Conceptualization; methodology; writing—original draft; writing—review and editing; funding acquisition. **A. Bradley Duthie**: Conceptualization; methodology; software; writing—original draft; writing—review and editing; supervision; funding acquisition. **Isabel Jones**: Conceptualization; writing—review and editing. **Daksha Patel**: Writing—original draft; writing—review and editing. **Nils Bunnefeld**: Conceptualization; methodology; writing—review and editing; supervision; funding acquisition.
